# The relationship between alertness and spatial attention under simulated shiftwork

**DOI:** 10.1038/s41598-020-71800-6

**Published:** 2020-09-11

**Authors:** D. Chandrakumar, J. Dorrian, S. Banks, H. A. D. Keage, S. Coussens, C. Gupta, S. A. Centofanti, J. M. Stepien, T. Loetscher

**Affiliations:** 1 Behaviour-Brain-Body Research Centre, Justice & Society, University of South Australia, Adelaide, SA Australia; 2 Appleton Institute, Central Queensland University, Health, Medical and Applied Sciences, Adelaide, SA Australia; 3 University of South Australia Online, Adelaide, SA Australia

**Keywords:** Human behaviour, Attention

## Abstract

Higher and lower levels of alertness typically lead to a leftward and rightward bias in attention, respectively. This relationship between alertness and spatial attention potentially has major implications for health and safety. The current study examined alertness and spatial attention under simulated shiftworking conditions. Nineteen healthy right-handed participants (M = 24.6 ± 5.3 years, 11 males) completed a seven-day laboratory based simulated shiftwork study. Measures of alertness (Stanford Sleepiness Scale and Psychomotor Vigilance Task) and spatial attention (Landmark Task and Detection Task) were assessed across the protocol. Detection Task performance revealed slower reaction times and higher omissions of peripheral (compared to central) stimuli, with lowered alertness; suggesting narrowed visuospatial attention and a slight left-sided neglect. There were no associations between alertness and spatial bias on the Landmark Task. Our findings provide tentative evidence for a slight neglect of the left side and a narrowing of attention with lowered alertness. The possibility that one’s ability to sufficiently react to information in the periphery and the left-side may be compromised under conditions of lowered alertness highlights the need for future research to better understand the relationship between spatial attention and alertness under shiftworking conditions.

## Introduction

Working around the clock is an integral part of a 24-h society. Approximately 15 to 30 percent of the working population comprise of shiftworkers^1, 2^. Shiftwork generally refers to work outside the typical 9am to 5 pm hours, such as morning, evening or night shifts. Due to these unusual schedules and poor sleep during the day, these workers often work at times when alertness is low^3^ and sleepiness is high^4^. Working outside conventional daytime hours may impact cognitive functioning^5^. There are also short-term effects, such as reduced productivity, impaired memory and increased safety risk (i.e., more error prone) during a night shift^6, 7^.

Shiftworkers have high levels of subjectively and objectively measured sleepiness during the night shift^4, 8^. This is due to shiftworkers being awake when their bodies are programmed to be asleep. A large proportion of shiftworkers work extended hours, and commonly report working consecutive shifts, leading to prolonged exposure to conditions which lower alertness^9^.

Sleepiness and low alertness levels are associated with higher risks for accidents, injury and error^10^, and therefore, shiftworkers are at a higher risk of being involved in incidents and accidents than non-shiftworkers^4^. For example, one study^11^ reported that medical errors in surgical residents increased by 22 percent with lowered alertness. Moreover, reduced efficiency in performance during night shift tends to be pronounced in the early hours of the morning (i.e., 2am–5am)^12^, coincident with a peak in incidents and accidents during this time^13^. Working a night shift also increases the likelihood of experiencing a near crash event on the drive home^14^.

One reason for the increased likelihood of crashes may be related to a narrowing of spatial attention with lowered alertness. Simulated monotonous driving performance in two studies revealed a deterioration in the ability to detect stimuli presented in the periphery, compared to central presentations, as driving time increased^15, 16^. This indicates that conditions that lower alertness, such as time-on-task, can narrow attention to the centre of the visuospatial field.

A growing body of literature link lower levels of alertness with a spatial shift of attention towards the right side^17^. This rightward shift in attention is characterised by slowed reaction times to left-sided stimuli^18^, left-sided stimuli misidentified as being on the right^19^, and omission of left-sided objects^20^. A study^20^ examined spatial attentional performance in hospital staff at two testing periods: one during a well-rested state (i.e., with an average of 7 h of sleep in the previous 24 h prior to testing), and another, during reduced alertness after working a double-shift (i.e., with an average of 3.55 h of sleep in the previous 24 h). The authors found a rightward shift in attention, indicating a neglect of the left side in the reduced alertness condition compared to the well-rested condition. Hence, these findings have safety implications for shiftworkers. For example, highway driving under high-speed conditions whilst operating a large vehicle, such as a truck, requires skilled motor and visuospatial ability^21^, and as truck drivers commonly commute during the night and early hours, this places them under monotonous driving conditions which can lower alertness^22^. Previous research suggests impaired performance resulting from lowered alertness is linked with vehicular accidents^23, 24^. Currently, the literature examining the spatial attention and alertness relationship is limited, highlighting the need for future research to explore the relationship between lowered alertness and spatial attention under shiftworking conditions, particularly whilst working the nightshift, when alertness is already reduced due to working at a time when the drive for sleepiness is at its greatest^3^.

Alertness can be separated into tonic alertness (referring to general alertness) and phasic alertness (referring to moment to moment alertness) and these can differentially influence spatial attention^25^. Tonic alertness can be measured via simple reaction time tasks which involve maintaining a state of preparedness to respond to stimuli, for a period of time^25^. Phasic alertness can be measured via cued-response tasks and can accelerate and enhance orienting towards a cued stimulus in healthy adults^26^. In neglect patients experiencing left-sided neglect, a reduction in this neglect under phasic alerting conditions is found^27^.

A dominant theoretical framework postulates a rightward shift in attention with declining alertness^28^. This is due to decreasing alertness resulting in an activation advantage in the left hemisphere, which directs attention to the right visuospatial field, and consequently leads to an attentional bias towards the right. Under alert conditions, the right hemisphere is slightly more activated, biasing attention towards the left due to contralateral attentional allocation. Therefore, changes in alertness should demonstrate corresponding changes in attentional biases, where high and low levels of alertness show a leftward bias and rightward bias, respectively.

The current study aimed to investigate the relationship between alertness and spatial attention under simulated shiftwork conditions. The main hypotheses were that (1) lowered alertness will correspond with a rightward bias in attention and high alertness will correspond with a leftward bias in attention, and (2) lowered alertness will be associated with a narrowing of attention.

## Method

### Participants

19 healthy non-shiftworking right-handed participants (11 males) aged between 18 and 39 years (M ± SD: 24.6 ± 5.3 years) were recruited from the general population. Initial eligibility screening involved gathering information regarding age, self-reported height/weight, smoking status, health status, and sleep patterns via telephone screening. Exclusion criteria were the following: presence of medical conditions, psychiatric disorders, sleep disorders, abnormal blood chemistries, habitual sleep duration < 7 h or > 9 h, BMI outside the normal to overweight range (18.5–27 kg/m^2^), regular medication use (oral contraception was allowed), drug and/or alcohol abuse, methamphetamine abuse, more than 2 h of structured high impact activity/exercise per week (to limit the extreme metabolic changes influencing results related to being sedentary in the lab for 7 days), food allergies, pregnancy, and any trans meridian travel in the 60 days prior to the study, or history of shiftwork, to ensure stable sleep/wake patterns and no circadian misalignment. If deemed eligible, following written informed consent to participate in the study, participants attended two further screening sessions in person to complete questionnaires regarding psychological and physical health, had a blood test completed, and wore an activity monitor for 7 consecutive days prior to the study commencing (Phillips Respironics Actiwatch, Murrysville, PA, USA). Participants also completed a 7-day sleep diary and a 4-day food diary to assess habitual sleeping and eating patterns, respectively. Female participants were only scheduled to participate in the luteal phase of their menstrual cycle, as confirmed by self-report, to control for changes in sleep quality, hormonal factors, and basal metabolism during the menstrual cycle^29^. Participants refrained from over-the-counter medications, caffeine, alcohol, napping, and were instructed to keep a strict habitual sleep schedule (22:00–23:00 to 06:00–07:00), which was verified by actigraphy and a subjective sleep diary during the 7 days prior to study commencement. Urine toxicology was performed to verify the absence of any illicit substances immediately prior to study commencement. These criteria are consistent with the laboratory’s previous sleep studies standard exclusion criteria^30, 31^. Handedness was assessed using the Flinders Handedness Survey (FLANDERS)^32^ and was not used as inclusion or exclusion criteria. Data collection for the present study took place within a wider study, which received approval by the University of South Australia Human Research Ethics Committee (#0000033621) and is registered with the Australian New Zealand Clinical Trials Registry (ANZCTR12615001107516). All procedures were in accordance with the National Statement on Ethical Conduct in Human Research.

### Procedure

**Figure 1:**
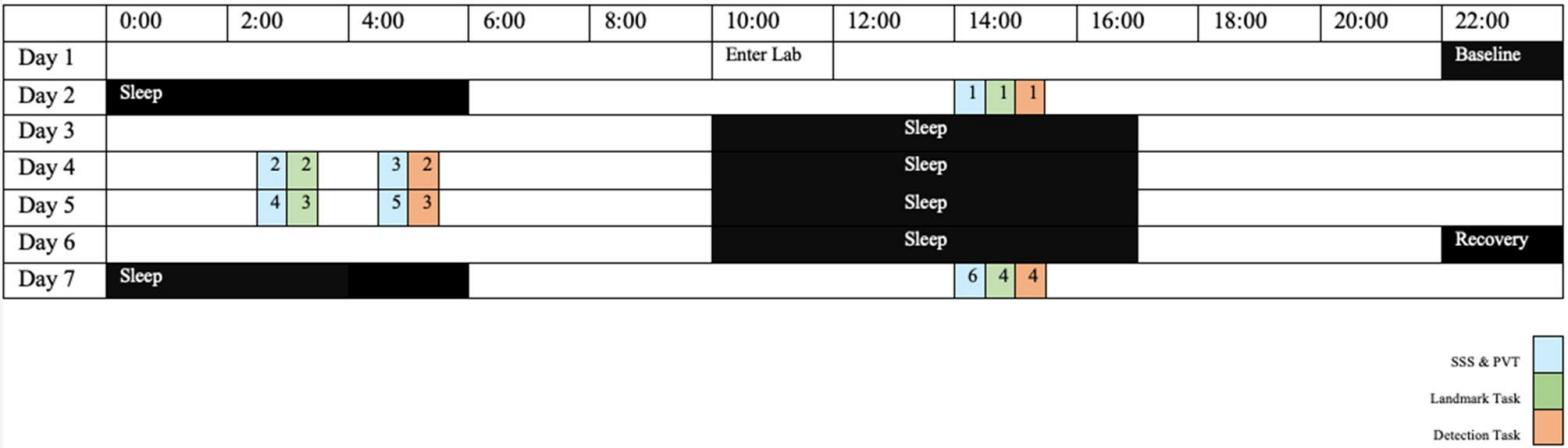
Simulated shiftwork protocol with the timepoints of the alertness (Psychomotor Vigilance Task [PVT], Stanford Sleepiness Scale [SSS]) and spatial attention (Landmark Task, Detection Task) measures outlined. The numbers correspond to the order of the tests.

The study was conducted at the Sleep and Chronobiology Laboratory at the University of South Australia. Participants spent six nights and seven days consecutively in a laboratory-controlled environment. The laboratory temperature was set to 22 ± 1 °C. Light intensity was < 100 lx at eye-level during wakefulness and was fixed at < 0.03 lx during sleep periods. On the first night, participants had an 8-h sleep opportunity, which followed a baseline day. Prior to the first night shift, participants were awake for 28 h to simulate the common pattern observed in shiftworkers prior to their first shift^4^. From days three to six, during the simulated night shifts, participants were given a 7-h daytime sleep opportunity on each day between 1000 to 1700 h. The last day (day 7) involved recovery sleep, where participants were given an 8-h sleep opportunity between 2200 to 0600 h to adjust their sleep/wake cycle from the shiftwork manipulation. Further information on the protocol can be found from a previously published study^33^. Data for the current study was collected at six different time points across six days. Refer to Fig. 1 for an outline of the testing protocol.

### Alertness measures

Two measures of alertness were employed in the current study, one assessing objective (behavioural) alertness, and the other assessing subjective (self-reported) alertness.

#### Psychomotor vigilance task (PVT)

A 10-min hand-held PVT device, which is highly sensitive to sleep loss was used to measure alertness in the current study. The PVT is regarded as a reliable and valid assay of alertness^34, 35^. The PVT required participants to respond as quickly as possible to the presentation of a visual stimulus (i.e., a red circle) which changes to a number as a timer counts from 0 to 500 ms. PVT performance at six time points were obtained in the current study: days two and seven at 1400 h, days four and five at 0300 h, and days four and five at 0500 h were examined. The PVT reciprocal of the mean reaction time (mean RRT) and lapses were used for analysis. Mean RRT was calculated as the mean of the reciprocal of the reaction time (1/RT) in milliseconds. A lapse was counted when reaction time was greater than 500 ms^36^. Lower mean RRT scores and higher lapses indicate poorer performance and lowered alertness.

#### Stanford sleepiness scale (SSS)

A 7-point rating scale was used to assess subjective sleepiness, ranging from 1 “*feeling active, vital, alert, or wide awake*” to 7 “*no longer fighting sleep, sleep onset soon; having dream-like thoughts*”^37^. The SSS has been found to predict performance on tasks that assess alertness, such as reaction time and vigilance tests^38, 39^. The SSS was used to assess subjective sleepiness throughout the protocol at the same six time points as the PVT (i.e., days two and seven at 1400 h, days four and five at 0300 h, and on days four and five at 0500 h). Higher SSS scores indicate higher levels of sleepiness.

### Spatial attention measures

Two measures assessing spatial attention, more specifically, one assessing judgement and the other assessing detection, were used in the present study. The judgment task entailed judgment of the spatial properties of lines, whilst the detection task required speeded responses to presented stimuli. Stimulus presentation for both tasks were presented using E-prime 2.0 software on a DELL desktop computer with a screen resolution of 1920 X 1080 and a diagonal screen size of 58 cm. Participants were seated approximately 60 cm from the centre of the screen, with their heads rested on a chinrest to maintain participants’ head position towards the centre of the screen.

#### Landmark task

**Figure 2:**
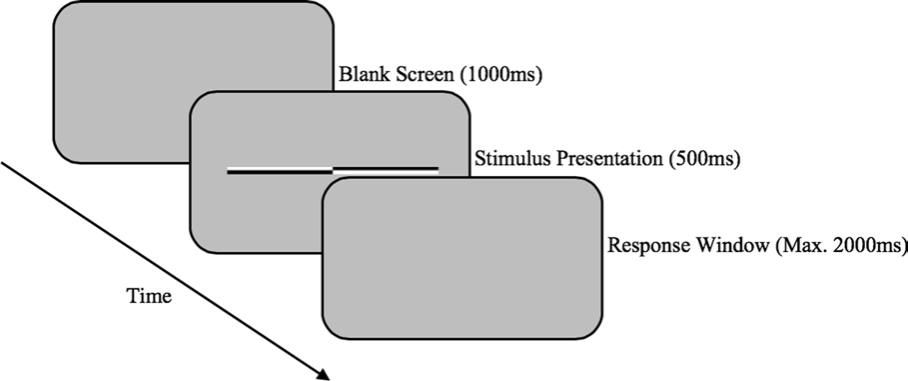
Landmark Task stimulus train. Prior to stimulus presentation, a blank screen was presented for 1000 ms. Stimulus presentation was 500 ms and the maximum response window was 2000 ms.

A commonly used spatial attention task, the Landmark task^18, 20, 40^ was employed in the current study. One hundred and eight vertically pre-bisected horizontal lines were presented against a grey background. Each pre-bisected horizontal line contained diagonally opposite pairs of black and white segments on each side of the vertical divider. The top segment on one side was white whilst the bottom segment of the same side was black, and vice versa for the opposite side. Side of longer segment (left/right), polarity (upper left black, upper left white) and deviation from the midline (0.5, 1, or 2 mm) differed between trials. Half the total number of stimuli were pre-bisected towards the left, and half the stimuli contained upper left black polarity. The factors side, polarity and deviation were counterbalanced between the 108 stimuli, such that each factorial combination was presented on nine occasions. Participants were instructed to indicate the side of the pre-bisected line that was longer, responding using the letter ‘z’ and ‘m’ on the keyboard to indicate left and right, respectively. Each trial started with a blank screen for 1000 ms followed by a stimulus presentation for 500 ms and a blank response window for a maximum of 2000 ms (see Fig. 2). If the participants responded within 2000 ms, the following trial was presented. However, if participants did not respond within the allocated response time window, the trial was presented again at a later stage, so each participant responded to 108 stimuli before task termination. The task lasted approximately eight minutes. Bisection bias on the Landmark Task was calculated as the number of right responses minus the number of left responses divided by the total number of trials for each participant^41^. Hence, negative and positive bias scores indicate a bias towards the left and right side, respectively. Landmark Task spatial bias on days two and seven at 1400 h, and days four and five at 0300 h were analysed.

#### Detection task

**Figure 3:**
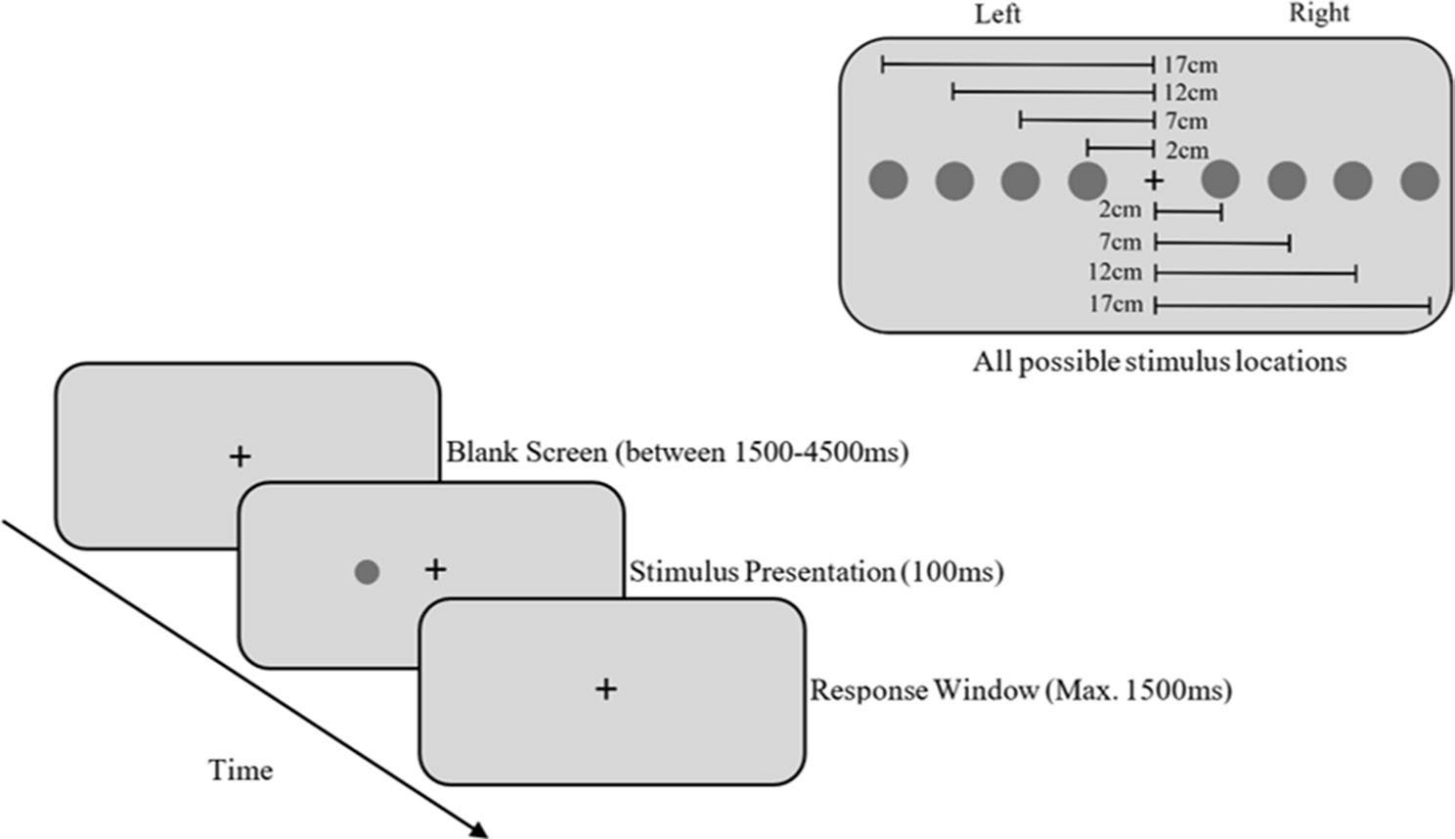
Detection Task stimulus train. Prior to stimulus presentation, a blank screen was displayed for a duration between 1500 to 4500 ms. Stimulus presentation was 100 ms with a maximum response window of 1500 ms.

Participants were presented with 5 mm circular dark grey stimuli at varying eccentricities on a light grey screen. Each circular stimulus was presented randomly at one of four eccentricities on each side of the fixation point at 2, 7, 12, and 17 cm from the centre of the screen with a height of 28 cm and width of 51 cm. A total of 120 stimuli were presented, with 15 stimuli at each of the eight screen positions. A fixation cross was presented throughout the trials. Each trial started with the presentation of the central fixation cross for a randomized presentation time ranging between 1500 to 4500 ms, followed by the stimulus presentation for 100 ms. The response window was set to a maximum of 1500 ms (see Fig. 3). The trial terminated once the participant responded or if no response was made after 1500 ms. If participants did not respond to a stimulus, that trial was considered an omission of response. If participants responded within 100 ms of stimulus presentation, this response was deemed an anticipatory response and removed from analysis. The task lasted approximately 12 min. Detection Task performance was assessed on days two and seven at 1400 h, and days four and five at 0500 h. The number of omissions (i.e., the trials that participants did not respond to stimuli) and the reaction time following stimulus presentation were used for analysis.

### Statistical analyses

#### Data analysis

Data was analysed using jamovi version 1.0.7.0^42^. Alertness measures (PVT & SSS) and spatial bias measures (bisection bias on the Landmark Task & reaction time and omissions on the Detection Task) corresponding to the time of testing (i.e., D2 1400 h, D4 0300 h, D4 0500 h, D5 0300 h, D5 0500 h, D7 1400 h) were used in analysis. Missing data was not imputed or corrected for in the analyses as mixed effects models allow for parameters to be estimated using available data^43^.

#### Alertness changes with time of testing

Three mixed effects models with ID as a random intercept were run to determine whether alertness changed across the different time points of the simulated shiftwork protocol. All three models included the categorical variable of time (D2 1400 h, D4 0300 h, D4 0500 h, D5 0300 h, D5 0500 h, D7 1400 h) as a fixed effect. The continuous variables PVT lapses, PVT mean reciprocal reaction time (mean RRT), or SSS were entered as the outcome variable for each model. F-values from fixed effects, degrees of freedom, and post hoc analyses are reported in Table 1. Holm corrections were used to account for multiple post hoc comparisons. Partial eta^2^ effect sizes were also calculated independently from the mixed effects modelling.

**Table 1:** F values, degrees of freedom, p values and holm corrected post hoc comparisons relative to time of testing, for each measure derived from the mixed effects models.

	Predictor	F	df	*p*	Post Hoc Comparisons, *p* < .05
**Psychomotor Vigilance Task (PVT)**					
PVT Lapses	Time	4.75	5, 90	< .001	D2 1400 h > D4 0500 h, D5 0500 h; D7 1400 h > D4 0500 h
PVT mean RRT	Time	7.61	5, 90	< .001	D2 1400 h > D4 0300 h, D4 0500 h, D5 0500 h; D7 1400 h > D4 0300 h, D4 0500 h, D5 0500 h
**Stanford Sleepiness Scale (SSS)**					
SSS	Time	39.2	5, 90	< .001	D2 1400 h > D4 0300 h, D4 0500 h, D5 0500 h, D7 1400 h; D4 0300 h > D4 0500 h, D5 0500 h; D5 0300 h > D4 0500 h, D5 0500 h; D7 1400 h > D4 0300 h, D4 0500 h, D5 0300 h, D5 0500 h
**Landmark Task**					
Bisection Bias	Time	2.75	3, 53.1	.052	–
**Detection Task**					
Reaction Time	Time	4.66	3, 546	.003	D2 1400 h > D4 0500 h
	Side	0.31	1, 546	.576	–
	Location	16.6	3, 546	< .001	2 cm > 12 cm, 17 cm; 7 cm > 12 cm, 17 cm
	Time*Side	0.67	3, 546	.572	–
	Time*Location	0.57	9, 546	.825	–
	Side*Location	0.12	3, 546	.950	–
	Time*Side*Location	0.35	9, 546	.956	–
Omissions	Time	17.3	3, 550	< .001	D2 1400 h > D4 0500 h, D5 0500 h, D7 1400 h; D7 1400 h > D4 0500 h, D5 0500 h
	Side	.001	1, 550	.977	–
	Location	29.5	3, 550	< .001	2 cm > 12 cm, 17 cm; 7 cm > 12 cm, 17 cm; 12 cm > 17 cm
	Time*Side	0.17	3, 550	.917	–
	Time*Location	0.60	9, 550	.794	–
	Side*Location	1.24	3, 550	.293	–
	Time*Side*Location	0.17	9, 550	.997	–

D = day. > corresponds to better performance relative to other levels of a factor (i.e., time or location). Locations 2 cm, 7 cm, 12 cm, and 17 cm correspond to distance from the centre of the screen.

#### Spatial attention changes with time of testing

Three mixed effects models with ID as a random intercept were run to investigate whether the Landmark Task bisection bias, and Detection Task reaction time and omissions related to time. All models had time as a fixed effect, and the continuous variables bisection bias, reaction time or omissions as the outcome variable for each model. Side and location were further predictors in the Detection Task models. F values, degrees of freedom, and post hocs comparisons with Holm corrections are reported in Table 1.

#### The alertness and spatial attention relationship

A series of mixed effect models were conducted to investigate the relationship between alertness and spatial attention during the simulated shiftwork protocol. For the Landmark Task, two linear mixed effects models with fixed effects of objective alertness, as measured by PVT lapses and PVT mean RRT, were conducted. PVT lapses and PVT mean RRT were predictors in separate models. Bisection bias was the outcome variable in these models. Another mixed effects model, with subjective alertness, as measured by the SSS, with the same time points as the previous model was conducted, and bisection bias as the outcome variable was run. ID was entered as a random intercept in all models.

For the Detection Task, six linear mixed effects models with fixed effects of objective alertness (i.e., PVT lapses, PVT mean RRT), or subjective alertness (i.e., SSS), were conducted to determine the relationship between alertness and spatial bias. The outcome variable was reaction time for three models and omissions for the remaining three models. Time, side of stimulus presentation (left/right), and location of stimulus presentation (2, 7, 12 and 17 cm) was entered as a fixed effect and ID was entered as a random effect in all models. Post hoc comparisons with Holm corrections were also conducted.

## Results

### Alertness changes with time of testing

**Figure 4:**
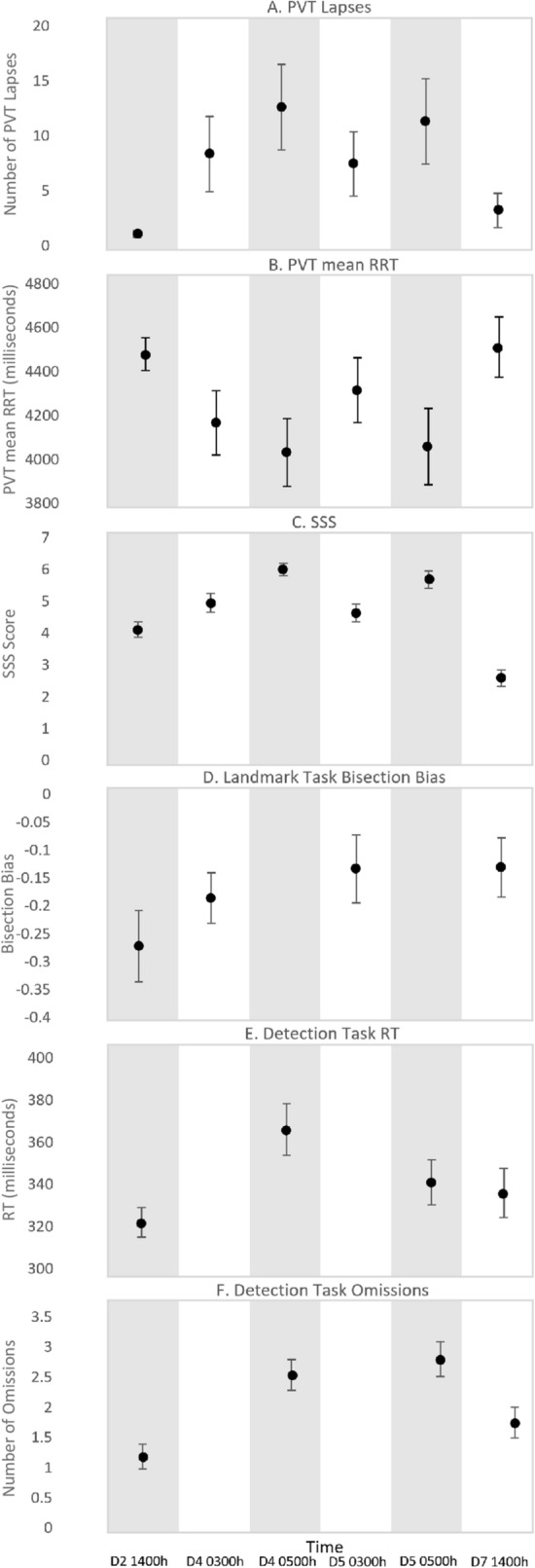
Alertness and spatial bias performance indicators change with day and time. Means and standard errors in alertness (PVT Lapses, PVT Mean RRT & SSS) and spatial bias (Landmark Task & Detection Task) throughout the protocol are depicted. A. High PVT lapses suggest poor performance. B. Low PVT mean RRT reflects poor performance. C. High scores on the SSS reflect high levels of sleepiness. D. Negative and positive scores on the Landmark Task bisection bias suggests a leftward and rightward bias, respectively. E. High Detection Task reaction times represent poor performance. F. High Detection Task omissions represent poor perfomance.

PVT lapses (beta = 7.32, 95% CI = 2.82 to 11.8), PVT mean RRT (beta = 4.26, 95% CI = 4.02 to 4.50), and SSS (beta = 4.66, 95% CI = 4.29 to 5.03) differed across time (Table 1). The calculated partial eta^2^ effect sizes were moderate for PVT lapses (0.21) and PVT mean RRT (0.30), and large for SSS (0.69). Figure 4 illustrates these changes in alertness over time, with alertness generally higher during the afternoon testing time points compared to the early morning testing time points.

### Spatial attention changes with time of testing

Spatial bias on the Landmark Task did not associate with time of testing (beta = −0.18, 95% CI = −0.27 to −0.09), refer to Table 1.

**Figure 5:**
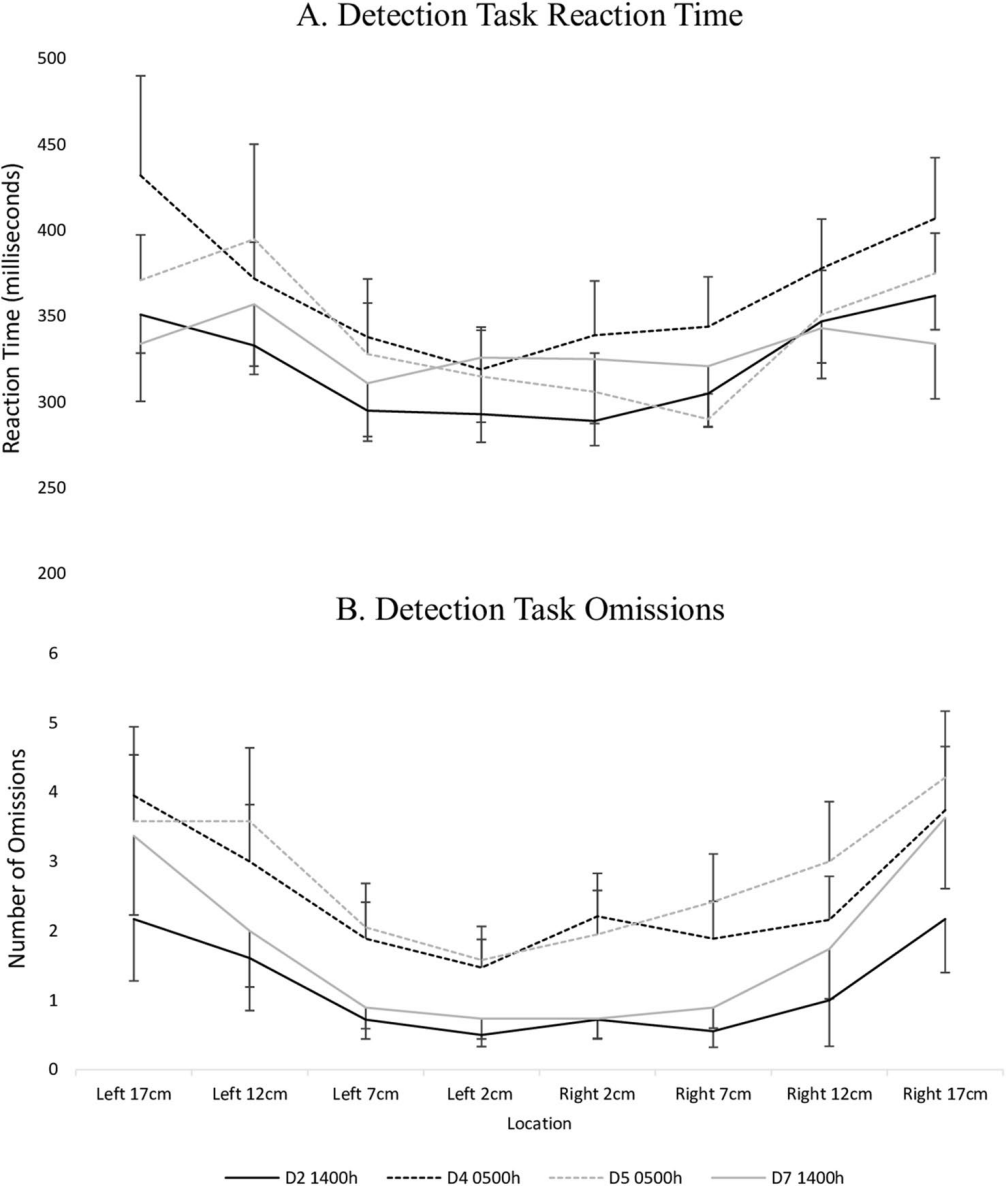
Performance in the periphery is worse than in the centre. Detection Task changes in reaction time (A), and omissions (B) with time, relative to stimulus location.

There was an effect of time of testing on Detection Task reaction time (beta = 343, 95% CI = 303–383) and omissions (beta = 2.06, 95% CI = 1.09 to 3.03), reflecting generally worse performance in the early morning compared to the afternoon. There was also a significant effect of location, such that performance was better when responding to stimuli closer to the centre compared to the periphery (Table 1). Figure 4 depicts the pattern of spatial attention performance with time of testing and Fig. 5 depicts the pattern of Detection Task performance relative to stimulus location across time of testing.

### The alertness and spatial attention relationship

#### Spatial bias in the landmark task

Objective measures of alertness: PVT lapses did not associate with bisection bias on the Landmark Task (beta = 0.001, CI = −0.01 to 0.004), and PVT mean RRT also did not associate with bisection bias (beta = −0.01, CI = −0.12 to 0.10).

Subjective measures of alertness: SSS did not associate with bisection bias on the Landmark task (beta = −0.003, CI = −0.03 to 0.03). Refer to Table 2.

**Table 2:** Fixed effect for the relationship between alertness and spatial bias*.*

Outcome	Predictors	F	df	*p*	Post Hoc Comparisons, *p* < .05
**Landmark Task**					
	PVT lapses	0.05	1, 68.5	.825	–
	PVT mean RRT	0.03	1, 70.2	.865	–
	SSS	0.03	1, 60.4	.861	–
**Detection Task**					
1. Reaction Time	PVT lapses	39.4	1, 580	< .001	–
	Side	1.14	1, 562	.286	–
	Location	11.0	3, 562	< .001	2 cm > 12 cm, 17 cm; 7 cm > 12 cm, 17 cm
	PVT lapses*Side	10.9	1, 562	< .001	–
	PVT lapses*Location	1.59	3, 562	.190	–
	Side*Location	0.01	3, 562	.978	–
	PVT lapses*Side*Location	0.91	3, 562	.438	–
2. Reaction Time	PVT mean RRT	73.5	1, 563	< .001	–
	Side	4.99	1, 562	.026	–
	Location	1.83	3, 562	.141	–
	PVT mean RRT*Side	4.72	1, 562	.030	–
	PVT mean RRT*Location	0.99	3, 562	.399	–
	Side*Location	0.29	3, 562	.831	–
	PVT mean RRT*Side*Location	0.26	3, 562	.858	–
3. Reaction Time	SSS	15.6	1, 570	< .001	–
	Side	0.14	1, 562	.713	–
	Location	1.03	3, 562	.378	–
	SSS*Side	0.36	1, 562	.547	–
	SSS*Location	0.34	3, 562	.798	–
	Side*Location	0.06	3, 562	.980	–
	SSS*Side*Location	0.11	3, 562	.955	–
4. Omissions	PVT lapses	66.3	1, 584	< .001	–
	Side	0.32	1, 566	.574	–
	Location	19.2	3, 566	< .001	2 cm > 12 cm, 17 cm; 7 cm > 12 cm, 17 cm; 12 cm > 17 cm
	PVT lapses*Side	1.49	1, 566	.223	–
	PVT lapses*Location	1.68	3, 566	.171	–
	Side*Location	0.37	3, 566	.778	–
	PVT Lapses *Side*Location	1.09	3, 566	.354	–
5. Omissions	PVT mean RRT	99.8	1, 571	< .001	–
	Side	0.0004	1, 566	.984	–
	Location	33.2	3, 566	< .001	2 cm > 12 cm, 17 cm; 7 cm > 12 cm, 17 cm; 12 cm > 17 cm
	PVT mean RRT*Side	0.33	1, 566	.568	–
	PVT mean RRT*Location	5.71	3, 566	< .001	–
	Side*Location	1.39	3, 566	.244	–
	PVT mean RRT *Side*Location	0.79	3, 566	.502	–
6. Omissions	SSS	32.1	1, 573	< .001	–
	Side	0.34	1, 566	.563	–
	Location	3.91	3, 566	.009	–
	SSS*Side	0.39	1, 566	.533	–
	SSS*Location	0.09	3, 566	.965	–
	Side*Location	0.10	3, 566	.960	–
	SSS*Side*Location	0.33	3, 566	.805	–

When predictors were categorical, all other categories were compared to the first category. Locations refer to distance from the centre of the screen (i.e., 2 cm, 7 cm, 12 cm, and 17 cm). “ < ” refers to direction of performance (i.e., 2 cm > 12 cm refers to better performance at location 2 cm than at location 12 cm).

#### Reaction time in the detection task

**Figure 6:**
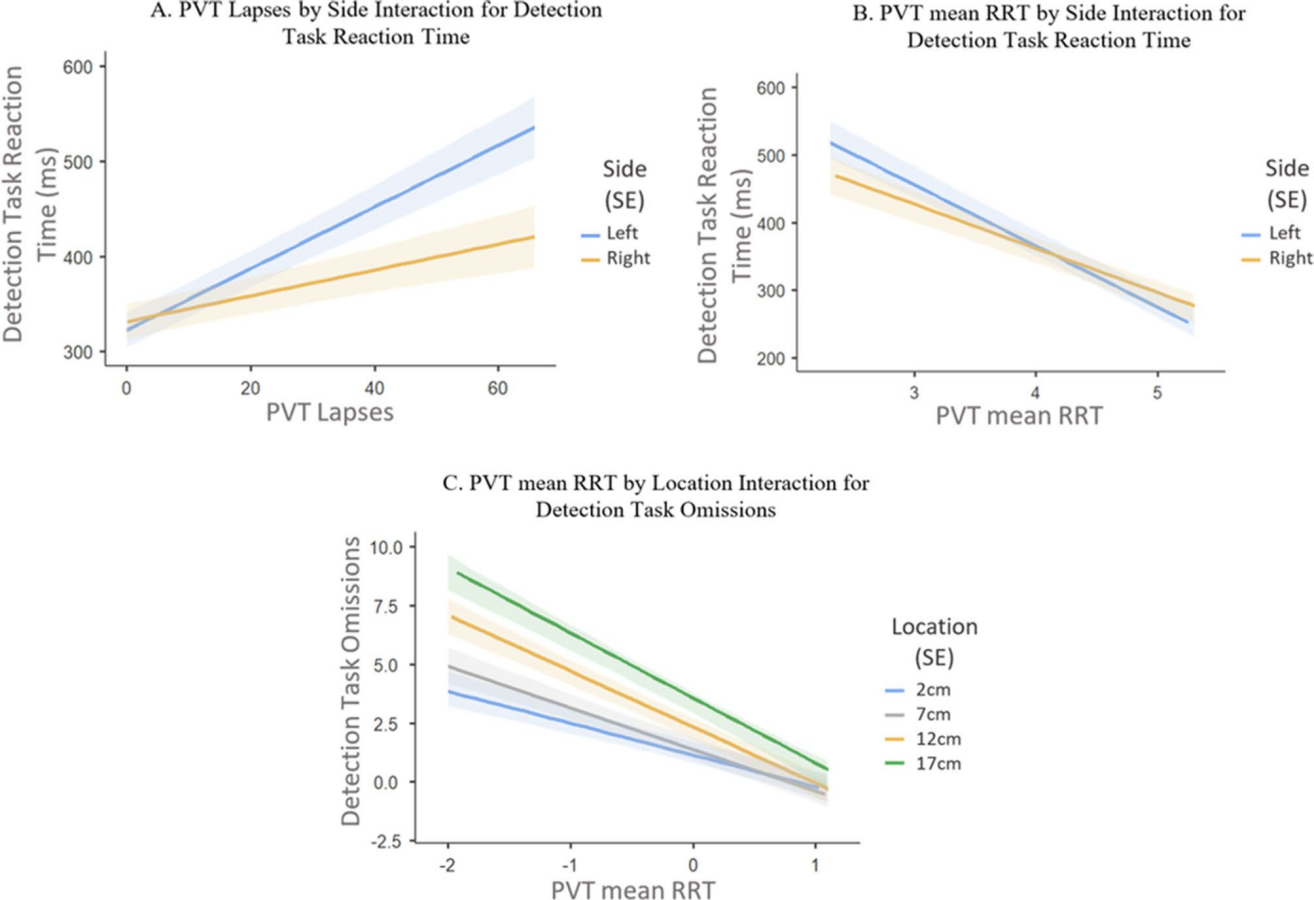
Mixed models data show Detection Task performance decreases with a decrease in alertness. A. Reaction time increases more strongly for left-sided stimuli with an increase in PVT lapses. B. Reaction time decreases more strongly for left-sided stimuli with an increase in PVT mean RRT (higher mean RRT scores reflect better performance). C. The further away the stimulus, the stronger the effect of PVT mean RRT on omissions. Shaded areas indicate standard error.

Objective measures of alertness: PVT lapses were related to reaction time on the Detection Task (beta = 2.29, CI = 1.58 to 3.01). That is, for every increase in a PVT lapse, there was a corresponding average increase in reaction time by 2.29 ms on the Detection Task. Location was a significant predictor of Detection Task reaction time (*p* < 0.001; refer to Table 2 for significant post hoc comparisons), indicating that reaction time to stimuli increased with an increase in stimulus distance from the centre. A PVT lapses by side interaction (*p* = 0.001) showed left-sided (compared to right-sided) stimuli to have a higher increase in reaction time with increasing lapses. Figure 6 (Panel A) portrays this interaction pattern.

PVT mean RRT was related to reaction time on the Detection Task (beta = −77.7, 95% CI = −95.5 to −60.0). That is, every increase in mean RRT corresponded with a decrease in reaction time by 77.7 ms on the Detection Task. There was a significant interaction of PVT mean RRT and side, such that the left side had a stronger increase in Detection Task reaction time with a decrease in PVT mean RRT (*p* = 0.030; Panel B in Fig. 6).

Subjective measures of alertness: Scores on the SSS also related to reaction time on the Detection Task (beta = 9.77, 95% CI = 4.92 to 14.6), suggesting that every unit increase in subjective sleepiness corresponds with an increase in reaction time by 9.77 ms on the Detection Task.

#### Omissions in the detection task

Objective measures of alertness: PVT lapses associated with Detection Task omissions (beta = 0.07, 95% CI = 0.06 to 0.09). That is, every increase in lapses related to a 0.07 increase in an omitted response on the Detection Task.

PVT mean RRT was predictive of Detection Task omissions (beta = −2.07, 95% CI = −2.47 to −1.66). The negative association shows every increase in PVT mean RRT to correspond with a reduction in 2 omissions. There was a significant PVT mean RRT by location interaction (*p* < 0.001) revealing that the further away the stimulus location, the stronger the effect of PVT mean RRT on omissions. Refer to Panel C Fig. 6.

Subjective measures of alertness: SSS associated with omissions on the Detection Task (beta = 0.32, 95% CI = 0.21–0.44), such that an increase in SSS corresponded with a 0.32 increase in an omitted response. Omissions were more likely in the periphery compared to the centre as demonstrated by a main effect of location (*p* = 0.009).

## Discussion

The current study’s findings provide some support for a narrowing of attention and a neglect of the left side with lowered alertness. We observed changes in PVT, subjective sleepiness, and response times and omissions on the spatial attention tasks over time that reflect typical circadian modulation. That is, lower levels of alertness in the night/early morning (0300 h, 0500 h) compared to the afternoon (1400 h)^4, 12^. There were also more omissions occurring in the periphery than the centre. However, while there were no significant spatial attentional shifts found in relation to time of testing, there was a relationship between shifts in spatial attention and PVT response times and lapses, which was our assay of behavioural alertness. As PVT response times and number of lapses increased, so did detection task response times, and this relationship was stronger for stimuli presented on the left side (consistent with the current model^28^), and for those in the periphery (illustrated in Fig. 6). Put differently, alertness is not reduced uniformly across the whole hemifield, rather a larger reduction is observed in the periphery especially in the left hemifield.

In contrast to the Detection Task, there were no associations between the spatial performances in the Landmark Task and the objective and subjective assays of alertness (see Table 2). The lack of associations stands in contrast to the findings from a previous study^20^. The authors examined Landmark Task performance in shiftworkers and reported a leftward bias with high alertness and a rightward bias with low alertness. However, in our study, participants were allowed up to 7 h of sleep per 24 h (data reported previously^33^), which in terms of sleep opportunity, is more consistent with the ‘well-rested’ condition in the previous study than their ‘sleepy’ condition, where participants achieved less than 4 h per 24h^20^.

The objective assays of alertness were only associated with the Detection task, but not the Landmark task. The reason for this observed dissociation is not clear. It cannot be ruled out that we were underpowered to detect subtle spatial shifts in the Landmark Task. The effect sizes for the Landmark Task and PVT assays of behavioural alertness suggests that the effects, although non-statistically significant and very small, were in the expected direction.

It is also possible that the lack of association in the Landmark Task is due to the fact that the two spatial tasks tap into different underlying mechanisms. A principal component analysis identified the Landmark and Detection tasks as distinct factors contributing to observed variances in spatial tasks^44^. The two tasks differ, for example, in the level they tap into the phasic alertness with higher levels for the Detection task due to the potentially alerting nature of the task’s stimulus presentation. They also differ in their cognitive processing demands with one task being a simple detection task and the other task requiring a judgment on spatial properties. However, the degree to which different tasks tap into different underlying spatial mechanisms is a matter of debate^45^ and our study was not designed to disentangle these mechanisms. Also, a recent meta-analysis did not find evidence that effect sizes for associations between alertness and spatial attention differ between tasks requiring speeded responses to stimuli (i.e., detection of stimuli) and judgement tasks such as the Landmark Task^17^.

The findings from the Detection Task revealed that there is a narrowing of attention to the centre from the periphery (with slower responses and more omissions to stimuli in the periphery) consistent with studies that generated alertness reductions through extended time-on-task and sleep restriction^15, 46^. The current study suggests that even under conditions of relatively common amounts of sleep, attention narrowing may be observed across circadian fluctuations in alertness, particularly as assessed by behavioural assay.

The relationship between spatial attention and alertness measures was stronger for PVT than for subjective alertness. In particular, PVT responses corresponded more strongly to performance decrements to left-sided stimuli (Table 2). This was not the case for subjective alertness, suggesting that behaviourally-assessed alertness is a closer reflection of shifts in spatial attention than self-reports.

PVT responses, but not time of the day, were associated with spatial biases. Individual levels of alertness at specific times can vary considerably between participants^47^. In contrast to PVT measures, individual differences are not captured with the relatively crude measure ‘time of the day’. The current study suggests that it is important to consider individuals’ alertness levels when investigating the relationship between alertness and spatial biases.

Overall, our findings highlight potential safety risks for occupations requiring a high level of visuospatial ability. While speculative at this stage, our results could have implications for shiftworkers who work unconventional hours. At times when behaviourally-assessed alertness is lower, individuals may be slightly biased towards the right-side of their environment and may be more likely to experience attentional narrowing, responding more slowly to, or missing, important information in their periphery. Under conditions, where shiftworkers are required to drive for work (e.g., truck drivers), or drive home after a night shift, their safety might be compromised. Past research into shiftwork and cognitive performance has revealed the detrimental effects of lowered alertness. For example, lowered alertness resulting from shiftwork is linked with an increased risk for accident and injury^48^. Based on our findings, researchers examining alertness should consider the influence of side of space when assessing safety and fatigue management in the future.

Our young, healthy participants in the study underwent a shiftwork manipulation to disrupt circadian timing in a tightly controlled laboratory environment, where they were relatively well-rested following sleep. In the field, shiftworkers tend to be chronically sleep deprived^3^, suggesting that narrowing of attention and a neglect of the left side could be worse in the real world where alertness levels are likely to fluctuate beyond those observed in our study, and may be influenced by many factors (e.g., worker age, health status).

The current study only examined six different time points within the simulated shiftwork protocol. This was a limitation of the study as investigating more time points might have given a clearer indicator of the relationship between spatial attention and alertness. Furthermore, testing both spatial tasks at the same timepoints, as opposed to 3am for the Landmark Task and 5am for the Detection Task, would have helped us assess the potential differential influence of the early morning hours on the spatial measures. Another limitation is that the participant sample comprised of only right-handed participants. This is a limitation as right- and left-handers have been found to show the opposite spatial attention and alertness pattern^17, 49^.

## Conclusion

To our knowledge, this study was the first to examine whether changes in alertness as a result of a simulated shiftwork protocol is associated with changes in spatial bias. The current study found some evidence for a slight neglect of the left side and a narrowing of attention with lowered alertness. The findings were not definitive and depended on the measures used to assess spatial attention and alertness. Given that our findings suggest the possibility that the ability to sufficiently react to information in the periphery and to the left side may be compromised with lowered alertness, this highlights the need for future research to better understand the relationship between spatial attention and alertness under shiftworking conditions.
